# Posterior Interosseous Neuropathy with Peripheral Dystonia: A Case Report

**DOI:** 10.5334/tohm.856

**Published:** 2024-04-22

**Authors:** Gohei Yamada, Takanari Toyoda, Eiichi Katada, Noriyuki Matsukawa

**Affiliations:** 1 Department of Neurology, Nagoya City University West Medical Center, Aichi, Japan; 2 Department of Neurology, Nagoya City University Graduate School of Medical Sciences, Aichi, Japan

**Keywords:** Dystonia, Pseudodystonia, Neuropathy, Posterior interosseus neuropathy

## Abstract

**Background:**

Posterior interosseous neuropathy is an uncommon cause of peripheral dystonia.

**Case Report:**

A 62-year-old man awakened and noticed right finger drop. A neurological examination revealed posterior interosseous neuropathy with dystonia-like finger movements. Abnormal movements were predominantly observed in the right thumb, ring finger, and little finger. Within 2 weeks, the muscle weakness in the right fingers had completely improved. However, a brief abnormal posture of the right thumb was persistent.

**Discussion:**

The residual abnormal posture of the right thumb may reflect pre-existing motor control abnormalities, which may have contributed to the onset of posterior interosseous neuropathy-associated peripheral dystonia.

## Introduction

Dystonia is characterized by persistent or repetitive muscle contractions and abnormal postures. Brain lesions in the basal ganglia, secondary motor cortex, and cerebellum can induce dystonia. Neuropathy-associated dystonia is a type of peripheral dystonia; however, the association between neuropathy and dystonia remains controversial. Abnormal posture and repetitive movement with additional clinical features not compatible with dystonia are thought to be pseudodystonia [1]. Here we report a case of posterior interosseous neuropathy (PIN) complicated by dystonia-like movements of the hand. In this case, a brief dystonia-like movement of the right thumb was observed despite clinical improvement of the neuropathy. We describe the phenomenology and mechanism underlying PIN-associated dystonia-like movements.

## Case Report

**Figure 1:**
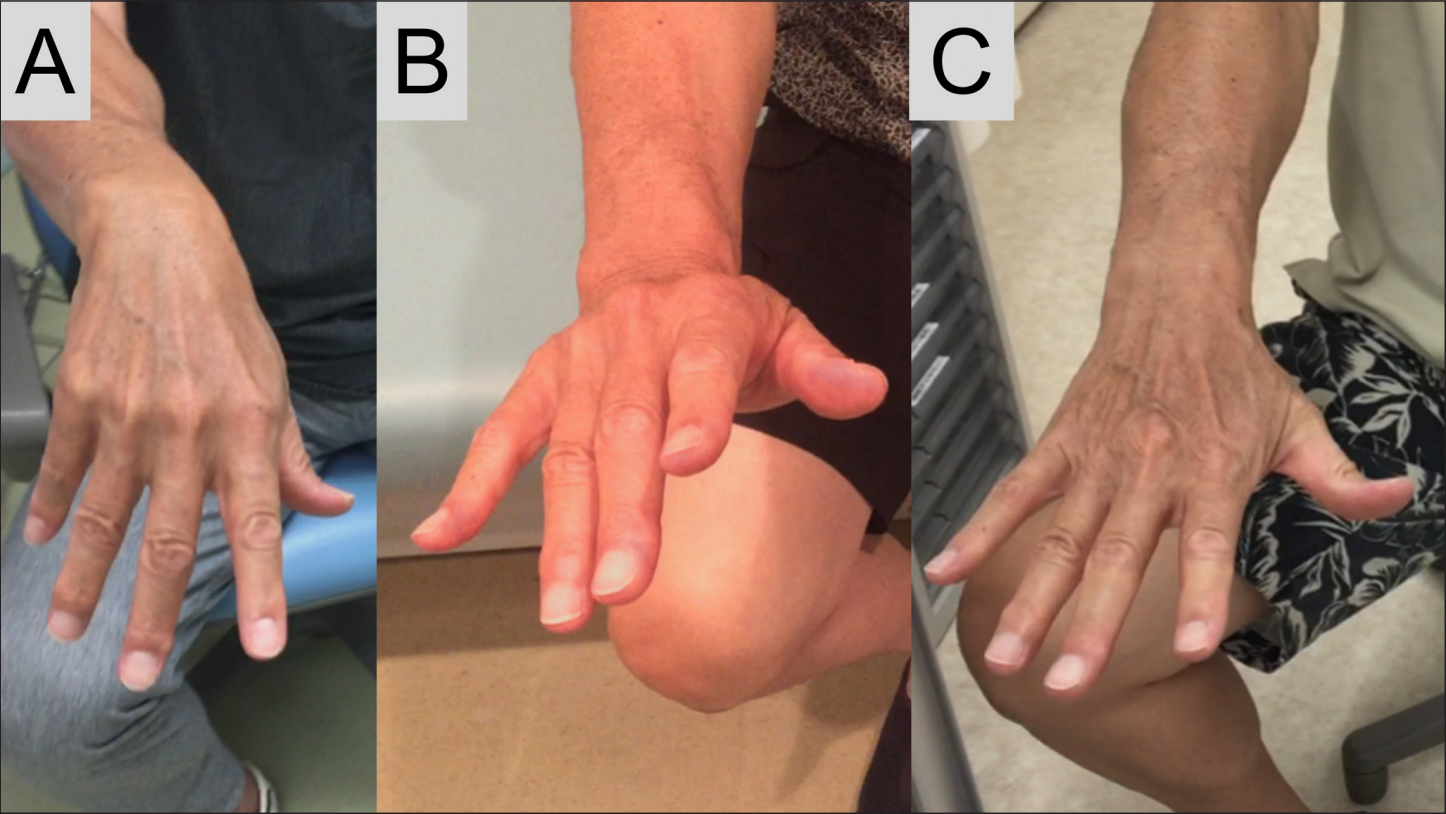
**Posture of the right hand.** Wrist and finger drop (A), incomplete “sign of the horn” (B), and normalization of hand posture (C). The “sign of the horn” represents an extension of the index and little fingers combined with weak or no extension of the middle and ring fingers.

A 62-year-old right-handed man with a past medical history of hypertension noticed a hand and finger drop and shaking finger movements on his right side upon awakening one morning (Figure 1A). The patient did not take any medication, had no clinical history of neurological disorders, including recurrent entrapment neuropathy, and had no family history of neuropathy or dystonia. Before falling asleep, he was able to move the individual fingers well without any involuntary motions. The patient seemed to develop muscle weakness and posture- or action-induced involuntary movements in the right hand almost simultaneously while sleeping. The patient came to our hospital seeking medical attention. On neurological examination, the strengths of the extensor indicis proprius (EIP), extensor digitorum communis (EDC), extensor pollicis brevis (EPB), abductor pollicis longus (EPL), extensor digiti minimi (EDM), and extensor carpi ulnaris (ECU) muscles were weak (Medical Research Counsil Scale for Muscle Strength [MRC] grade 2/5) with sparing of the brachioradialis, biceps, triceps, deltoid, and median- and ulnar nerve-innervated muscles on the right side. The muscle weakness was distributed in the innervated region of the right posterior interosseous nerve. No sensory impairment was detected. The deep tendon reflexes in the upper and lower extremities were decreased. The Trömner and Hoffman reflexes were absent. Involuntary flexion of the ring and little fingers and jerky movement of the wrist were observed on the right side while the patient was extending the bilateral arms forward with all fingers extended and abducted (Video 1). When the patient repeated extension and flexion of the right fingers, brief involuntary flexion of the right little finger was observed. At rest, subtle quick movement was observed in the right fingers. The patient had slight difficulty extending and flexing the right individual fingers sequentially. While the patient flexed the index and middle fingers sequentially with the thumb flexed, involuntary extension and abduction of the thumb occurred briefly on the right side (Video 1). There was no sensory trick or null point to suppress involuntary movements.

**Video 1:** **Neurological examination at the initial visit.** Abnormal postures of the thumb, ring finger, and little finger, jerky movement of the right wrist, and subtle quick movement of the fingers at rest are visible on the right side.

**Figure 2:**
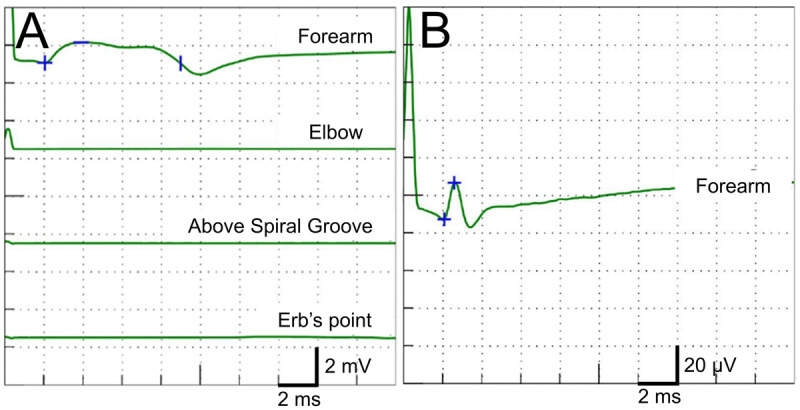
**Nerve conduction study of the right radial nerve.** Compound muscle action potential **(A)** and sensory nerve action potential **(B)**.

Magnetic resonance imaging of the brain showed no causative lesions. Computed tomography of the neck showed no occupying lesions within the cervical canal or around the brachial plexus. A nerve conduction study revealed decreased amplitude of the right radial nerve compound muscle action potential (CMAP) recorded from EIP and a conduction block between the forearm and elbow (Figure 2A). There was no significant difference in the amplitude, distal latency, nor duration of the radial nerve CMAP between the right and left sides (1.1 mV and 1.4 mV, 2.1 ms and 2.4 ms, and 6.9 ms and 6.6 ms, respectively). As the muscle strength of the left EIP was normal, we considered that the low value of the radial EIP CMAP on the left side had no pathological significance. On the right side, a small axonal loss may be present with a conduction block in the posterior interosseous nerve. Considering the subsequent rapid recovery, a conduction block between the right forearm and elbow appeared to be the major pathological finding. The sensory nerve action potential (SNAP) of the superficial radial nerve was normal (Figure 2B). There were no abnormal findings in the median nerve CMAP and F wave recorded from the abductor pollicis brevis and median nerve SNAP or the ulnar nerve CMAP and F wave recorded from the abductor digiti minimi and ulnar nerve SNAP. Although the cause of the entrapment was unclear, the right elbow joint might have been transiently compressed by his body during sleep. The patient did not take sedative medications or alcohol the night before the possible compression. The patient was diagnosed with PIN.

On the following day, the muscle weakness was slightly ameliorated. The muscle strength of the EDC was MRC grade 2, while that of the EIP, EPB, EPL, EDM, and ECU was MRC grade 3. Thus, an incomplete “sign of the horn” was observed while the patient extended all of the fingers (Figure 1B) [2]. Involuntary flexion of the right ring and little finger persisted (Video 2). However, the range of motion of the involuntary movements was decreased compared with that on the previous day. Involuntary extension and abduction of the thumb occurred briefly while the patient flexed the index and middle fingers sequentially with the thumb flexed (Video 2). Postural jerky movement of the right wrist and subtle quick movement of the right fingers at rest did not occur. Within 2 weeks after symptom onset, the patient completely recovered with conservative management (Figure 1C). Involuntary extension and abduction of the right thumb during flexing the right fingers was persistent but asymptomatic (Video 3). Because the patient did not undergo a neurological examination before visiting our hospital, the timing of the onset of residual involuntary movement of the right thumb was unclear.

**Video 2:** **Neurological examination on the following day**. Abnormal posture of the right ring and little fingers is mild compared with that seen on the previous day.

**Video 3:** **Neurological examination one month later**. The neurological examination is unremarkable except for the brief abnormal posture of the right thumb during flexion of the right fingers.

## Discussion

Herein, we reported a case of PIN associated with abnormal finger postures and jerky movement of the wrist. We considered these abnormal movements to be a type of peripheral dystonia, as trauma and neuropathy are the major causes of peripheral dystonia [3,4]. Radial nerve palsy, lower brachial plexopathy, median neuropathy, ulnar neuropathy, and thoracic outlet syndrome are reported potential causes of peripheral dystonia [5,6,7]. The association between peripheral dystonia and PIN in two musicians was previously reported, but the detail of peripheral dystonia was not described [8]. In the present case, peripheral dystonia was demonstrated for the first time in a non-musician patient with PIN.

The strict discrimination between dystonia and pseudodystonia in patients with peripheral dystonia is sometimes challenging [1]. In the present case, the abnormal finger postures and jerky movement of the wrist were considered pseudodystonia rather than dystonia for the following reasons. First, there were some associated findings in the affected body region, which is a key feature of pseudodystonia [1]. Temporal association between PIN and the onset of abnormal finger postures in the same distribution was implied. Subtle quick movement of the right fingers at rest may reflect peripheral nerve hyperexcitability. Second, the absence of a sensory trick suggests pseudodystonia. Sensory trick is found in approximately 70–80 percent of patients with dystonia [9]. Third, abnormal finger postures appeared acutely following the onset of PIN. Dystonia usually presents insidiously, while pseudodysytonia usually presents acutely. Fourth, there was the absence of specific trigger, such as handwriting in writer’s cramp, which supported the diagnosis of pseudodystonia. However, we could not exclude the possibility of dystonia. The sensory trick is uncommon in secondary dystonia. The time of onset of abnormal posture of the right thumb was unclear and may not be associated with PIN, furthermore, it was difficult to confirm that all abnormal finger postures had an acute onset. Abnormal finger postures were mobile and evoked by hand posture and action. While patients with pseudodystonia present with fixed postures at rest and during action, those with dystonia present with mobile postures during action [1].

A definite association between neuropathy and pseudodystonia is yet to be established. However, there are several possible mechanisms which may underlie the development of neuropathy-associated pseudodystonia. Four cases of pseudodystonia secondary to multifocal motor neuropathy have been previously reported [10]. A potential cause of pseudodystonia is presumed to be hyperexcitable peripheral nerves with ectopic discharges due to the site of conduction block. In these four cases, abnormal movements began in the distribution of peripheral nerves with motor conduction block, and subsequently spread to other nerve-innervated muscles. In the present case, abnormal finger postures and jerky flexion movement of the wrist were generated from the activation of the median and ulnar nerve innervated muscles. The widespread muscle involvement suggests that the central nervous system may be modulated through peripheral feedback from the injured peripheral nerve-innervated muscles. Peripheral deafferentation is another potential cause of pseudodystonia. Peripheral deafferentation due to the anesthetic block of the limb transiently leads to disinhibition at the cortical or subcortical level and rapid modulation of motor output within minutes [11,12]. This rapid disinhibition is associated with the hyperexcitability of the corticospinal tract. Although sensory impairment was absent on neurological examination and nerve conduction studies, sensory information from the posterior interosseous nerve-innervated muscles may be altered, and lead to an almost simultaneous onset of PIN and pseudodystonia.

However, neuropathy alone cannot explain the low frequency of neuropathy-associated pseudodystonia. Most patients may have unrecognized motor control abnormalities [7]. Although its appearance was brief, the patient had residual abnormal posture of the right thumb despite clinical improvement of the PIN. The residual abnormal finger posture might have no direct association with PIN and may reflect the underlying susceptibility to a movement disorder. A potential explanation is that surround inhibition may have already been slightly reduced prior to the onset of PIN, and complications of the PIN may lead to the failure of sensory-motor integration and exacerbation of abnormal finger postures. We did not conduct a nerve conduction study again after the clinical improvement of the PIN, and subtle demyelination may be present in the posterior interosseous nerve and may contribute to abnormal posture of the right thumb.

In conclusion, the association between PIN and peripheral dystonia in a non-musician patient was first reported here. PIN overlapped with motor control abnormalities may play a crucial role in the development of peripheral dystonia.
